# Histocompatibility of Light-Curing Composites Used in Pediatric Dentistry in Human Oral Fibroblast Cells

**DOI:** 10.30476/dentjods.2022.92358.1631

**Published:** 2023-03

**Authors:** Milad Arab-Nozari, Ehsan Zamani, Mehdi Evazalipour, Banafsheh Soleimani, Ali Jorbonian, Azam Nahvi

**Affiliations:** 1 Dept. of Toxicology and Pharmacology, Faculty of Pharmacy, Ayatollah Amoli Branch, Islamic Azad University, Amol, Iran; 2 Dept. of Pharmacology and Toxicology, School of Pharmacy, Guilan University of Medical Sciences, Rasht, Iran; 3 Dept. of Pharmaceutical Biotechnology, School of Pharmacy, Guilan University of Medical Sciences, Rasht, Iran; 4 Dental Research Center, Mazandaran University of Medical Sciences, Sari, Iran; 5 Dept. of Pediatrics, Faculty of Dentistry, Mazandaran University of Medical Sciences, Sari, Iran; 6 Dentistry Student, Dental Research Center, Mazandaran University of Medical Sciences, Sari, Iran

**Keywords:** Composite, Fibroblast, MTT

## Abstract

**Statement of the Problem:**

Tooth-colored composites are used to repair caries lesions and other dental defects, particularly in anterior regions in children. Although a wide range of composites is using, little attention has been paid to the important indicators such as biological profiles or products released from these materials.

**Purpose:**

The current study aimed to compare the histocompatibility and cytotoxicity of light-curing resin used to repair children's teeth with different brands (3M, DenFil, and Opallis) in curing times of 20 and 40 seconds in human oral fibroblast cells (HGF1).

**Materials and Method:**

In this *in vitro* study, Three types of flow composites (3M, Opallis, and DenFil), all at A2 shade, were used. The composites were at 4×2mm with separate exposure times of 20 and 40 seconds. MTT test was used to determine the cytotoxicity of composites on oral fibroblast cells. This test is based on the conversion of tetrazolium bromide to a purple compound known as formazan that its color intensity can be evaluated using the ELISA. The higher intensity of the color reveals the higher survival rate of the mitochondria, which indicates less toxicity. One-way variance analysis and unpaired t-test were used to compare the cytotoxicity of each brand in two conditions of 20 and 40 seconds of curing. Statistical significance was considered when *p*< 0.05.

**Results:**

3M and Opallis composites were significantly reduced vitality of cells compared to control group in both 20s and 40s curing status. While DenFil brand did not show marked cytotoxicity. In each brand, there are no significant deference between 20s and 40s curing times.

**Conclusion:**

Histocompatibility depends on the type of composite resin. In the current study, DenFil brand showed the highest histocompatibility, followed by 3M and Opallis.

## Introduction

Tooth-colored composites are used to restore tooth defects and other dental defects, particularly in the anterior regions [ 1 ]. These materials, like other dental materials, in addition to having good physical and chemical properties, should have appropriate tissue compatibility and do not cause damage or toxicity. Moreover, they should not induce inflammation or immune response [ 2 ]. This feature has been given a high weight in approving a dental substance, for instance by American Dental Association (ADA) and Food and Drug Administration (FDA). When using these materials to restore dental cavities, especially for those that are near the gums, their contact with gums is almost unavoidable [ 3 ]. 

On the other hand, due to their long contact with adjacent tissues like periodontium, the probability of cytotoxicity due to the presence of different chemical compounds is high. Therefore, it is necessary to investigate the toxic effects of monomers in these substances [ 3 ].

Some *in vitro* studies reported that polymerized resins have no harmful effects on humans. Unfortunately, the oral environment is not appropriate for polymathic reactions of these substances, and some studies showed that even cured compounds release chemical compounds [ 4 - 5 ]. Among different methods developed for cytotoxicity evaluation, the dimethylthiazol-2-yl diphenyltetrazolium bromide (MTT) test is more appropriate due to its high speed and low cost as well as higher sensitivity [ 6 ]. Developed by Mossman, this test is based on investigating the effect of a compound on the survival of mitochondria, which is based on the conversion of a salt called tetrazolium bromide to a purple compound known as formazan by an enzyme (succinate dehydrogenase) that its color intensity can be evaluated using the ELISA [ 6 ]. The higher intensity of the color reveals the higher survival rate of the mitochondria, which indicates less toxicity [ 7 - 8 ].

Histocompatibility indicates the function ability of a material in certain conditions in the presence of an appropriate host response [ 9 ]. The need to use materials with histocompatibility indicates the necessity of cytotoxicity studies. Basically, *in vitro* studies are intended to evaluate cytotoxicity or genetic toxicity of substances [ 4 , 9 - 10 ]. Bationo *et al*. [ 9 ] assessed cytotoxicity of dental and orthodontic light-cured composite resins (Clearfil ES-2, Clearfil ES Flow, Filtek Supreme XTE, Grengloo, Blugloo, Transbond XT, and Transbond LR) and reported that the cell viabilities were between 85 and 90%. In another study, Franz *et al*. [ 10 ] compared the cytotoxicity of packable and non-packable composites in one-way and bilateral curing and found that two-way curing composites had less cytotoxicity. Although a wide range of composites is available, little attention has been paid to the important indicators such as biological profiles or products released from these materials. Due to the high demand for operative dentistry, it is necessary to investigate the histocompatibility of currently available materials.

The current study aimed to compare the histocompatibility and cytotoxicity of three types of light-curing resin used to repair children's teeth with different brands (i.e., 3M, DenFil, and Opallis) in curing times of 20 and 40 seconds in human gingival fibroblast (HGF1).

## Materials and Method

### Composite's preparation

In this *in vitro* study, three types of flowable composites (3M, Opallis and DenFil), all at A2 shade, were used (Table 1).

**Table 1:** The profile of applied composites (3M, Opallis, and DenFil

Manufacturer	Country	Brand name	Type	Shade
3M ESPE	USA	3M	Microfill	A2
Vericom	South Korea	DenFil	Microhybrid	A2
FGM	Brazil	Opallis	Microfill	A2

To facilitate the use of composites and access to a standard and desirable size in terms of the quantity and weight used for composite restorations, each composite was placed separately in circled forms made of Teflon in dimensions of 2mm thick and 4 mm in diameter [ 11 ]. The required amount of light cure composites were placed into the desired mold and cured by using LED light curing unit (850mW/cm2,woodpecker, Guang Dong, China) in two separate times of 20 and 40 seconds (to investigate the effect of radiation time) with a distance of 2mm. Then, we used a Mylar matrix strip on the surface to limit oxygen inhibition [ 12 ]. To maintain sterile conditions, all stages of composite preparation were carried out in a laminar hood equipped with a UV lamp. Three samples were taken from each composite group, and each of the prepared samples was placed in a separate 96-plate well. Subsequently, 200μl of Dulbecco's Modified Eagle's Medium (DMEM) solution was added to the medium, followed by incubation for 24h at 37oC (CO2%5). We also considered a control group containing only the medium.

### Cell Preparation

HGF-1 cell line was purchased from Pastor Institute of Iran (Tehran), as prepared vials. All cells were transferred to the fresh culture medium (containing 10% FBS and penicillinester), followed by incubation for 48h at 37oC. Then, the cells were counted by trypan blue staining. This color cannot breach live cells, but it can enter the dead cells and turn them blue, which helps to count the cells.

### MTT Test

Initially, 10000 live cells were poured into each of the 96- plate well. Then, 100ml of the medium in which the composites were immersed, was added to the wells, followed by incubation for 48h at 37oC (CO2 %5) with the cells. Afterward, the MTT solution was prepared from the dissolution of yellow tetrazolium bromide powder in phosphate-buffered saline (PBS) with a concentration of 5mg/ml. This solution is highly sensitive to light and therefore was wrapped around the container and placed in the refrigerator. Then, 50μL of MTT solution was added to both control and intervention wells, followed by incubation for 4 h. Then, we removed the MTT solution, and 50μL of diluted dimethyl sulfoxide (DMSO) solution (250μL DMSO in 5ml of DMEM) was added. Later, DMSO was removed by pipette, and the adsorption intensity of dyed cell colonies was read by ELISA reader (Bio Tek, USA) at 570 nm wavelength [ 13 ]. Eventually, the cell viability percentage of samples was calculated according to the following equation [ 13 ]: 

Cell viability percentage = (Absorbance of each sample/ Absorbance of control) × 100

### Statistical Analysis

Data were analyzed using SPSS version 16 software. One-way variance analysis was used to compare the cytotoxicity of different brands. In addition, the unpaired t-test was used to compare cell cytotoxicity at 20 and 40 seconds of curing. Statistical significance was considered when *p*< 0.05.

## Results

**Figure 1:**
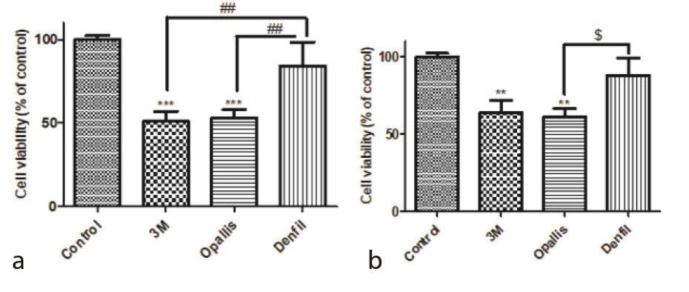
**a:** Effects of different composites on the cell viability at 20 seconds. The results were reported as Mean±SD (N=3). *** Significant difference with the control group (*p*< 0.001). ## Significant difference with the DenFil group (*p*< 0.01). ###significant difference with the DenFil group (*p*< 0.001), **b:** Effects of different composites on the cell viability at 40 seconds The results are reported as Mean±SD and all experiments were performed at triplet, *** Indicating a significant difference with the control group (0.001) $: indicates a significant difference with the DenFil group (*p*< 0.01)

**Figure 2:**
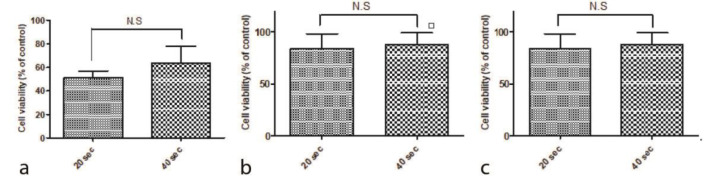
**a:** Effects of 3M composite on the cell viability at 20 and 40 seconds, **b:** Effects of Opallis composite on the cell viability at 20 and 40 seconds. C:Effects of DenFil composite on the cell viability at 20 and 40 seconds, **c:** The results are reported as Mean±SD and all experiments were performed at triplet (N.S: non-significant)

In this study, cytotoxicity and survival percentage of human oral fibroblast cells after exposure with three different composites (DenFil, Opallis, and 3M) were investigated at times of 20 and 40 seconds (Table 2). As shown in Figure 1, exposure of cell lines with 3M and Opallis composites for 20 seconds resulted in significant decline in cell survival compared to the control group (*p*= 0.0004 and *p*= 0.0005 respectively), while the DenFil composite did not cause any significant effect (*p*= 0.1539). In comparison of all three brands, the cytotoxicity of 3M and Opallis was significantly higher than the DenFil (*p*= 0.0047 and *p*= 0.0068 respectively), but had no significant effect compared to each other (*p*= 0.9892). According to Figure 1b, which is based on the curing time of 40 seconds, both 3M and Opallis brands significantly decreased the survival percentage of HGF-1 (*p*= 0.0063 and *p*= 0.0040 respectively), but the DenFil did not show significant toxicity compared to the control group (*p*= 0.4521). In comparison of three brands, only the Opallis brand showed significant changes compared to DenFil brand (*p*= 0.0302) and in other modes, no significant difference was observed. The cell survival percentage of composite brands at curing times of 20 and 40 seconds are provided in Figures 2. As can be seen, the survival percentage of HGF-1 was decreased in curing time of 20 seconds compared to that of 40 seconds, which these changes were not statistically significant for any of the brands (*p*= 0.3055 for 3M, *p*= 0.8605 for Opallis and *p*= 0.7510 for DenFil brand).

**Table 2:** Evaluation of fibroblasts cell survival in different composites

Groups	Curing time (seconds)	Cell survival mean (% of control)	SD
Control	20	100	2.26
	40	100	2.26
3M	20	51.01	5.88
	40	63.83	13.86
Opallis	20	53.05	4.79
	40	61	5.51
DenFil	20	84.08	14.16
	40	88.15	10.98

## Discussion

This study investigated the possible cytotoxic effect of three different brands dental composites in oral fibroblast culture medium using MTT method. There are no study has yet investigated the tissue compatibility of flowable composites with different brands. Despite the wide range and variety of composites used, and high demand for operative dentistry, it is necessary to investigate the histocompatibility of existing composites [ 14 ]. 

Resin-matrix of many of the currently available composites is based on the Bisphenol-A glycerin dimethacrylate (Bis-GMA) or urethane dimethacrylate (UDMA). Bis-GMA monomer has the highest cytotoxicity, followed by UDMA, triethylene glycol dimethacrylate (TEGDMA), and 2-hydroxyethyl methacrylate (HEMA) monomers. The release of these resin compounds may cause gingival tissue damage. At high concentrations, most monomers can intervene in protein synthesis and induce apoptosis. Geurtsen *et al*. [ 15 ] reported that Bis–GMA and UDMA are highly toxic to fibroblast cells. In contrast, the camphorquinone has relative cytotoxicity.

TEGDMA and HEMA monomers are cytotoxic in gingival cells and are probably responsible for allergies reactions. In addition, unbound monomers can induce bacterial growth, particularly the microorganisms, which are involved in the formation of dental caries [ 16 ].

Even completely light-cured, HEMA is not fully linked; a part could be released and therefore, an allergic reaction can occur. HEMA can be able to pass through the dentin tubules and end up in pulp tissue. Furthermore, the potential toxic reactions of various associated monomers seem to be greater than toxicity of each monomer when studied individually [ 9 ]. Although these findings cannot be generalized to dental clinics, careful use of these materials is necessary, particularly for children who are more sensitive compared to adults [ 14 ]. 

In this experiment, the MTT test was used to investigate the viability and activity of cells, which is the most common test used for evaluating cytotoxicity. It is worth noting that the sensitivity of this test is higher than other currently available tests [ 17 ]. 

The present study aimed to investigate monomer release after 24 hours of exposure. In addition, previous studies [ 18 - 19 ] showed that the highest cytotoxicity effect of composites is in the first 24 hours. Triton *et al*. [ 18 ] and Tell *et al*. [ 19 ] investigated self-cure composites used in orthodontics and showed that immediately after mixing and final polymerization, these composites had high toxicity, which decreases over time but still there are levels of toxicity. 

The current study investigated the effect of radiation duration (20 and 40 seconds), and a negative association was found, which was not statistically significant. In addition, according to the findings, increased volume of the filler and declined size of filler particles can improve biocompatibility.

D'Souza *et al*. [ 5 ], in a study on the cytotoxicity of light cure composites, concluded that the compounds cured for 40 seconds had lower cytotoxicity than those cured for 20 seconds. This issue refers to the importance of light cure duration [ 5 ], which can be attributed to the lower conversion of monomer-to-polymer and more release of toxic monomers when curing time is insufficient.

Franz *et al*. [ 10 ] investigated the cytotoxic effect of packable and non-packable composites in one-way and bilateral curing and showed that two-way curing composites had less toxicity. The level of monomer release affects the cytotoxicity of composites, which depends on several factors like light curing time, light penetration power, radiation intensity, and concentration of light initiators. These factors contribute to the full polymerization of these composites [ 20 ]. 

The sufficiency of conversion rate of monomers has a crucial role in their biocompatibility. Caughman *et al*. [ 20 ] reported a negative association between cytotoxicity and monomer release with light curing duration. In addition, it is proved that increased filler content and decreased filler particle size is associated with improved biocompatibility [ 5 ].

The composites used in this study have different combinations of methacrylate matrix, particulate size, type, and volume of filler (Table 1). In our current study, we compared the cytotoxicity of these three composite brands on human oral fibroblast cells. Our data showed that 3M and Opallis composites were significantly reduced vitality of cells compared to control group in both 20s and 40s curing status. On the other hands, DenFil brand did not show significant cytotoxicity in comparison to the control group. 

In this study, DenFil composite with cell viability of 84% had less cytotoxicity, while other composites with cell viability of nearly 50% (3M: 51% and Opallis: 53%) had more cytotoxicity. The lower cell viability can be attributed to the higher percentage of un-bonded monomers in 3M and Opallis composites, which were released in the culture medium. 

In addition, DenFil composite, according to the manufacturer, is a micro-hybrid composite with a high volume of inorganic fillers. Hybrid composites have 75-85% inorganic filler weight to improve the desired physical and mechanical properties [ 21 ]. In general, due to the relatively high content of inorganic filler, the percentage of resin was reduced, and as a result, less toxicity of this composite was observed in the present study. However, 3M and Opallis composites are microfilm composites with lower weight percentage and more resin content; therefore, the viability of the cells was lower for these composites. Sadeghian *et al*. [ 14 ] investigated the tissue compatibility of three types of resin composites including two types of self-curing composite (3M and Fantastic-Zardent) and a light cure composite (3M-Transbond XT). They reported that Fantastic showed lower cytotoxicity than 3M. In addition, they noted that the 3M light cure composite did not show toxicity. 

Schweiker *et al*. [ 21 ] studied the cytotoxic effect of four types of composites by MTT method with a proximity time of 24 hours and reported the cytotoxicity of these composites from highest to lowest as Solitaire2, Tetric Ceram, Dyract AP, and Definite, respectively.

Schedule *et al*. [ 22 ] studied six types of light-curing composites and reported that all types of composites showed some degree of cytotoxicity that is negatively associated with the incubation time. They also noted that the combination of composite and bonding showed more toxicity than composites. 

The observed difference in results of cell culture studies indicates that changes in the chemical structure of composites and changes in filler and monomer ratio have an important impact on the release of elements and toxicity levels. In addition, these studies have differences concerning the factors such as cell type, tested materials, incubation time, type of curing device and curing time of composites, which were different in these studies. MTT test evaluates cell metabolism or function, which is based on determining the quantity of biochemical activity of cells and the activity of some cell enzymes [ 23 ], but the results of this study did not indicate cell death. Hence, further studies are needed to achieve results that are more definitive.

According to the findings, it is suggested that during clinical application of composites, contact with gingival tissue should be prevented as much as possible. Protective equipment such as rubber dam is useful to achieve this purpose. In addition, over-contouring and invasion of restorative composites into the gingival space should be avoided as much as possible [ 24 ]. 

## Conclusion

This study showed that composite resins used in pediatric dentistry have different biocompatibility standards, which depends on composition and percentage of unbound monomers. In the current study, DenFil (microhybrid) showed the highest histocompatibility, followed by 3M and Opallis (Microfill). 

## Acknowledgment

This study was extracted from dentistry student thesis of Ali Jorbonian and supported by a grant from Mazandaran University of Medical Sciences, Sari, Iran.

## Conflict of Interest

The authors declare that they have no conflict of interest.
